# Medical History from the Earliest Times

**Published:** 1891-11-07

**Authors:** E. T. Withington


### Medical History from the Earliest Times.

By E. T. Withington, MA.., M.B. Oxon.

### I.—INTRODUCTORY.

Dr. Oliver Wendell Holmes, in a recent address, urges his hearers not to look with contempt on their old medical books; "the débris of broken systems and exploded dogmas," he continues, "form a great mound, a Monte Testaccio of the shards and remnants of old vessels which once held human beliefs. If you take the trouble to climb to the top of it, you will widen your horizon, and in these days of specialised knowledge your horizon is not likely to be any too wide." Now that the period of purely professional education has been prolonged, the tendency to this narrowness of view is likely to increase, and no better antidote could possibly be found than the study of medical history, a subject which makes us acquainted with the most diverse forms of thought, and brings before us every phase of civilization. The neglect of this study, especially in England, has often been noticed, and seems due to two causes; first, that those who might write histories of medicine are more practically employed,—some, perhaps, in the nobler task of making history,—secondly, and chiefly, that there is no demand for such works. The power and originality of modern medicine, the vigour and success with which it has opposed the scientific methods of observation and experiment to the old reverence for system and authority, tend to create a contempt for medical history, and a half-confessed feeling that we are the men, and knowledge was born with us, or at least with our fathers.

But the study has other advantages besides that of counteracting the narrowness of specialism. If we may no longer assert with Hippocrates that new discoveries can only be made by following out old tracks, medical history will at least warn us from paths which lead nowhere, or but to barren deserts of speculation; and though we shall meet with quacks, charlatans, and other relatives of that brisk lad, Ignorance, who came from the land of Conceit, we shall more often become acquainted with men whose characters we may well emulate, whose lives were devoted to the benefit of their fellows, and to a noble even if fruitless search for Truth. Nor is this study to be commended to the medical profession only, but to educated men in general, and that quite apart from its claims as an important and interesting section of the history of human development. Many who are too apt, when in health, to cast unmerited reproach and ridicule upon medical science, and too ready, when health fails, to run after every new "system" or fresh form of quackery, would thus learn something of the nature of medical problems. They would become acquainted with the vast difficulties against which medicine has had to contend; with the labours of those great men who have by slow degrees advanced the bounds of knowledge; in short, to quote once more our medical poet, with the history of "a noble profession, which for more than two thousand years has devoted itself to the pursuit of the best earthly interests of mankind, always assailed and insulted from without by such as are ignorant of its infinite perplexities and labours, always striving in unequal contest with the hundred-armed giant who walks in the noonday, and sleeps not in the midnight, yet still toiling, not merely for itself and the present moment, but for the race and for the future."

An attempt will be made in ensuing articles to take a brief survey of medical history, giving special prominence to those parts of it which teach such lessons as are above indicated. The subject is conveniently divided into three periods, of which the first may be called the instinctive, empiric, or, from its characteristic theory of disease, the demonic epoch of medicine. Under this head we shall consider the earliest forms of the healing art, supplementing our scanty knowledge of prehistoric medicine by what is known of its practice among uncivilized races. The medicine of the ancient East, and its early developments in Greece, will be classed here, though in Egypt and India especially we shall find ample evidence of a much higher stage of knowledge.

The great name of Hippocrates introduces a new era, that of Greek medicine, which forms the second, and most extensive division of our subject. After tracing the origin of clinical observation to this great reformer, and of anatomy and physiology to the fostering care of the Greek kings of Egypt, we shall see how the followers of Hippocrates, like those of his contemporary, Socrates, split up into a number of sects or schools, some of which entirely renounced his authority. Three of these sects, the Dogmatic, the Empiric, and the Methodic will attract our special attention, and we shall find them in the second century, A.D., producing their three greatest representatives, Galen, Sextus Empiricus, and Soranus the Methodist. We shall notice how the elaborate system of Galen, while eclipsing all the other schools, was itself obscured for a time by those vast revolutions in European history due to the triumph of Christianity, the invasion of the Barbarians, and the separation of the east and western divisions of the Roman Empire. In the Dark Ages which followed, the study of Greek medicine was taken up by another branch of the human family, and in the West at least was for a time classed among those *studia Saracenorum*, or Arabic studies, which were held to be closely allied to necromancy, and fraught with danger to the souls of the orthodox. Soon, however, in the story of the school of Salerno, we shall find Greek medicine returning to the West under Arab influences, somewhat modified in form, enriched with a larger armoury of drugs, and provided with a new attendant science in the infant chemistry. We shall next see how, at the revival of learning, this Græco-Arabic medicine came into collision with its genuine Greek original, and how the vast fabric of Galenism, thus divided against itself, and attacked from without by the new school of anatomists, and by free spirits such as Paracelsus, finally gave place to a new epoch. For two thousand years Greek medicine had ruled supreme, and though we can now afford to despise its humoral pathology, we shall, I think, find that even Galenism was superior to many of the systems it supplanted, and to some which have succeeded it, and that the Greek medicine as a whole was not unworthy of that wonderful people who gave it origin.

Our third period dates from the rise of a physiology founded no longer on speculation but on fact, and begins with that great discovery which has given the name of Harvey an immortality of reputation. But it was the betrothal rather than the marriage of Medicine and Science which was then celebrated. Chemistry, physics, physiology were still too young to take their proper position as helpmates to the healing art, and the attempt to hasten the union by seizing upon isolated sciences or scientific theories gave birth to a new set of schools and systems, chemical, mechanical, vitalistic, presenting many curious analogies to the old Greek ones. Happily their duration was shorter; great men continued the work of Harvey, and, at the beginning of the present century, medicine under Bichat and Laennec became, at least in some of its departments, scientific; systems, with one exception—homœopathy—disappeared, and medicine, like all other sciences, entered upon a period of unparalleled progress.

